# The isolation and characterization of CTC subsets related to breast cancer dormancy

**DOI:** 10.1038/srep17533

**Published:** 2015-12-03

**Authors:** Monika Vishnoi, Sirisha Peddibhotla, Wei Yin, Antonio T. Scamardo, Goldy C. George, David S. Hong, Dario Marchetti

**Affiliations:** 1 Biomarker Research Program Center, Houston Methodist Research Institute, Houston, TX; 2 Department of Pathology & Immunology, Baylor College of Medicine, Houston, TX; 3 Department of Investigational Cancer Therapeutics, The University of Texas MD Anderson Cancer Center, Houston, TX; 4 Department of Molecular & Cellular Biology and The Dan L. Duncan Cancer Center, Baylor College of Medicine, Houston, TX

## Abstract

Uncovering CTCs phenotypes offer the promise to dissect their heterogeneity related to metastatic competence. CTC survival rates are highly variable and this can lead to many questions as yet unexplored properties of CTCs responsible for invasion and metastasis *vs* dormancy. We isolated CTC subsets from peripheral blood of patients diagnosed with or without breast cancer brain metastasis. CTC subsets were selected for EpCAM negativity but positivity for CD44^+^/CD24^−^ stem cell signature; along with combinatorial expression of uPAR and int β1, two markers directly implicated in breast cancer dormancy mechanisms. CTC subsets were cultured *in vitro* generating 3D CTC tumorspheres which were interrogated for biomarker profiling and biological characteristics. We identified proliferative and invasive properties of 3D CTC tumorspheres distinctive upon uPAR/int β1 combinatorial expression. The molecular characterization of uPAR/int β1 CTC subsets may enhance abilities to prospectively identify patients who may be at high risk of developing BCBM.

Tumor relapse is a significant clinical problem which is particularly relevant in breast cancer where patients are asymptomatic because disseminated cells appear to become dormant, are undetectable by clinical tools, and residual disease remains dormant for periods longer than 20 years^1^,^2^. Uncovering phenotypes of circulating tumor cells (CTCs), the “seeds” of intractable metastasis, offers the promise to dissect CTC heterogeneity in relation to metastatic competence, to predict biomarker assessment, and to significantly improve monitoring and treatment of cancer^3^,^4^,^5^,^6^. Further, transcriptional profiles of CTCs directly isolated from breast cancer patients are distinct from ones of breast cancer cell lines that are widely used for drug discovery, a finding which raises issues regarding the appropriateness of using cell lines to model breast cancer therapy^7^,^8^. However, there is little knowledge of the molecular properties of CTCs and their biology. For example, it is still unknown whether and how CTCs differ in their capacity to circulate while maintaining metastatic potential. Rates of CTC survival can be highly variable, lasting less than a few hours in some patients but in the order of decades in others^9^,^10^. This can lead to many questions associated with as yet unexplored mechanisms of patient-derived CTCs responsible for mechanisms associated with tumor dormancy, along with their properties and functionalities.

Breast cancer is the second most common cancer to metastasize to brain and the prognosis of patient diagnosed with brain metastasis remains poor^11^,^12^. Further, adjuvant and systemic therapy drugs with a poor ability to penetrate the blood-brain barrier are associated with a higher risk of patients associated with breast cancer brain metastasis (BCBM)^12^. New targeted therapies, eg, to HER2, may be linked to antitumor effects on brain metastasis and improved survival. Lastly, there is no current ability to predict the likelihood of BCBM onset^12^.

We have previously reported the discovery of CTCs that do not express the common carcinoma epithelial cell adhesion molecule (EpCAM-negative CTCs) and possess high competence to generate BCBM in xenografts^13^. We posited that specific EpCAM-negative CTCs subpopulations, shed from the primary tumor and found in the circulation, avoid organ arrest with extreme efficiency by the concomitant presence of stem cell and quiescence properties. The molecular switch to differentiate quiescence in malignant CTCs depends on the cross-talk between CTCs and the tumor microenvironment. Of note, previous studies have established the presence of two neoplastic markers, urokinase plasminogen activator receptor (uPAR) and integrin β1 (int β1) promoting tumor cell growth and proliferation when they interact with the extracellular brain microenvironment^14^,^15^. However, the loss of uPAR and int β1 expression strikingly reduces proliferative signals causing a shift from an invasive or metastatic to a dormant state, and directly implicating these two biomarkers in mechanisms of tumor cell dormancy *in vivo*^1^,^2^,^14^,^15^.

Here, we report the isolation of subsets of EpCAM-negative breast cancer CTCs containing stem-cell properties (CD44^+^/CD24^−^) by multiparametric flow cytometry with a combinatorial uPAR and int β1 expression and their abilities to grow long-term *in vitro*. Second, we characterized CTC subsets possessing six cell surface expression markers (CD45^−^/EpCAM-negative/CD44^+^/CD24^−^/uPAR^+/−^/int β1^+/−^) to determine the expression profiling of candidate genes related to breast cancer and embryonic stem-cell pathways and demonstrate their tumor origin as putative CTCs. Third, we investigated CTC subsets for cell adhesion, proliferation properties, and for subset abilities to generate *in vitro* 3D CTC tumorspheres (3D-spheroids) and invade into extracellular matrix. Lastly, we sorted uPAR and int β1 CTCs at single-cell level by employing the DEPArray^™^ platform and performed mutation analyses to reveal unique genomic signatures of uPAR/int β1 CTC subsets.

In summary, we provide first-time evidence for the isolation of intra/inter-patient EpCAM-negative, uPAR/int β1 CTCs subsets with distinct capabilities for long-term *in vitro* growth; along with mechanistic link of these CTC subsets to cell adhesion, proliferative and invasive properties relevant to BCBM onset.

## Results

### Subsets of CTCs isolated from breast cancer patients grow *in vitro* and are capable of generating CTC tumorspheres

**Figure 1:**
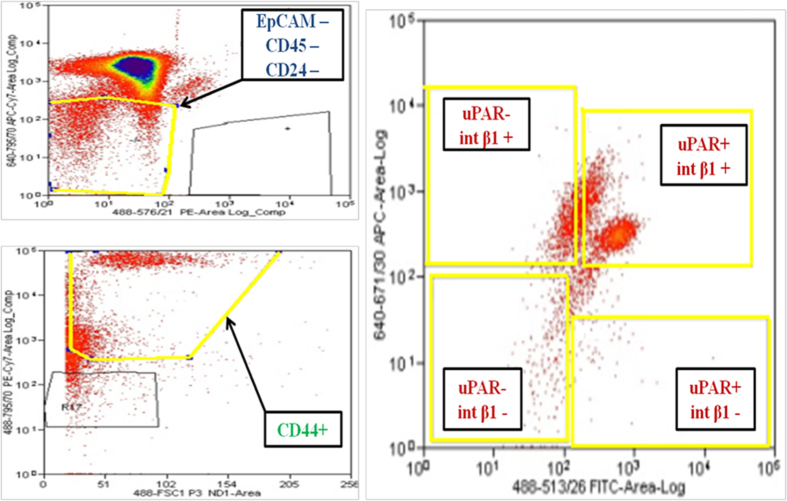
Multiparametric flow cytometry of PBMCs capturing uPAR/int β1 CTC subsets. Breast cancer PBMCs were first sorted applying gating parameters to select for DAPI^−^ (4′, 6-diamidino-2-phenylindole)/EpCAM^−^/CD45^−^/CD44^+^/CD24^−^ cells. Cells were then subsequently sorted to obtain uPAR/int β1 subsets containing combinatorial expression of these markers. Antibodies used for flow cytometry and cell sorting were: anti-human CD45-APC-Cy7 (Biolegend, cat # 304015, 1:50 dilution), mouse anti-human EpCAM-PE CD326 (eBiosciences, cat # 12-9326-71, 1:40 dilution), anti-human CD24-PE ML5 (Biolegend, cat # 311106, 1:20 dilution), anti-human CD44-PE-Cy7 IM7 (Biolegend, cat # 103030, 1:20 dilution), mouse anti-human uPAR (CD87)-FITC (AbD Serotec cat # MCA2506FT, 1:10 dilution), anti-human int β1 (CD29)-ApC TS2/16 (Biolegend, cat # 3030008, 1:50 dilution). Cells were confirmed to be CTCs by performing RT-PCR, immunoflurescence and genotyping arrays. Representative images are shown.

**Figure 2:**
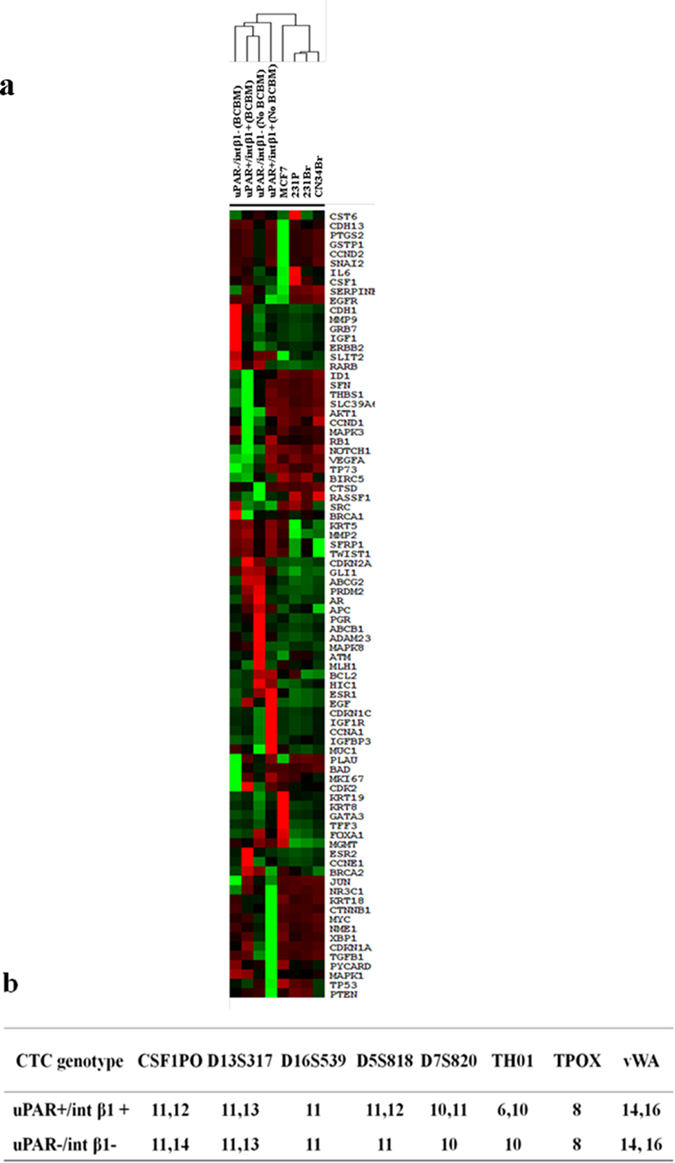
FACS-sorted CTC populations derived from primary breast tumors. (**a**) Breast cancer gene expression array profiling of FACS-enriched CD45^-^/EpCAM-negative/CD24^−^/CD44^+^/uPAR^+/−^/int β1^+/−^ CTC subsets derived from BCBM and no BCBM patients. mRNAs were amplified by REPLI-g WTA single cell kit (Qiagen) followed by real-time PCR analysis. Ct values and fold expression were calculated by online RT^2^ PCR profiler array data analyses software version 3.5 (http://pcrdataanalysis.sabiosciences.com/pcr/arrayanalysis.php) (Qiagen). Heat map and clustergram analyses were generated by online software Treeview and Cluster (Eisen lab, University of California, Berkeley). BCBM, Breast Cancer Brain Metastasis; **(b)** STR DNA fingerprinting of FACS-sorted CTC subsets derived from BCBM patients have unique profiles over cell lines available to NCI databases.

To establish whether subsets of CTCs isolated from the same patient and possessing a combinatorial uPAR/int β1 expression could be expanded in culture, we analyzed blood from patients’ peripheral blood mononuclear cells (PBMCs) employing multi-parametric flow cytometry analysis (FACS, ARIA IID, BD Biosciences™) by selecting DAPI^−^/ CD45^−^/EpCAM-negative/CD44^+^/CD24^−^/uPAR/int β1 expression markers to capture four combinatorial subsets (uPAR^+^/int β1^+^, uPAR^+^/int β1^−^, uPAR^-^/int β1^+^, uPAR^−^/int β1^−^) respectively (Fig. 1). Blood samples were obtained from 38 breast cancer patients clinically diagnosed with (n = 21) or without brain metastasis (n = 17) (Table 1 & Supplementary table S1). Next, to prove the tumor origin of DAPI^−^/CD45^−^/EpCAM-negative/CD24^−^/CD44^+^/uPAR/int β1 cells as putative CTCs, we performed transcriptome analysis of 83 breast cancer candidate genes present in human breast cancer real-time PCR (RT^2^-PCR) profiler arrays (Qiagen). Heat map and hierarchical clustergram analyses of flow-cytometry derived cells and their comparison with human breast cancer cell lines was performed. They showed the presence of gene expression patterns (CST6, CDH13, PTGS2, GSTP1, CCND2 and SNAI2) specific to breast cancer in isolated CTCs (Fig. 2a)^14^. Conversely, gene expression profiling of CTCs subsets derived from patients with and without clinically diagnosed BCBM have their unique profile (ID1, SFN, THBS1, CCND1, AKT1, MAPK3, RB1 and others) were not consistent with established BCBM cell lines [MDA-MB231Br (231Br for brevity) and CN34Br] (Fig. 2a).

Second, we carried out comprehensive genotyping analyses on CTC subsets derived from BCBM patients either with the presence or absence of uPAR/int β1 expression. We applied short tandom repeat (STR) DNA fingerprinting (16 loci). These CTC subsets possessed unique STR DNA fingerprinting profiles and were distinct from ones employing cancer cell lines from available databases (http://bioinformatics.istge.it/clima/) and from each other (Fig. 2b).

**Figure 3:**
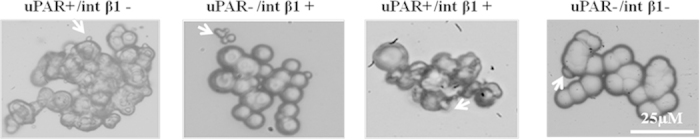
Morphological characterization of CD45^−^/EpCAM-negative/CD44^+^/CD24^−^/uPAR^+/−^/ int β1^+/−^ CTC subsets cultured as *in vitro* 3D CTC tumorspheres. FACS-enriched CTC subsets derived from breast cancer patient cultured in Mammocult media^™^ (StemCell Technologies, Inc.). CTC subsets grew as *in vitro* 3D CTC tumorspheres using stem cell and non-adherent conditions. White arrows indicated small-vesicle-like cells. Images were taken at 40X by phase contrast microscopy (Zeiss, Inc.). Representative images are shown.

Third, we interrogated CTC subsets by their abilities to be viable and expand *in vitro*. We were able to grow CTCs as non-adherent 3D CTC tumorspheres regardless of whether they were derived from BCBM vs no BCBM patients and independent of uPAR/int β1 expression (uPAR^+^/int β1^+^, uPAR^−^/int β1^−^, uPAR^+^/int β1^−^ and uPAR^-^/int β1^+^). We were able to grow CTC subsets under normal aerobic conditions (37 °C with 5% CO_2_) using 1% soft agar on 6-well tissue culture plates^17^ (Fig. 3). Of note, lowering O_2_ levels to hypoxic conditions (37 °C with 3–4% CO_2_) did not significantly affect CTC subsets growth. CTCs subsets were passaged using 0.25% trypsin (Gibco Life Technologies, Inc.). However, they tended to grow and expand as clusters (CTC tumorspheres) and dissociated only as singlets or paired cells. CTC-generated tumorspheres grew *in vitro* having two distinct cell sizes. We classified CTCs <5 μM diameter as small CTCs and >5 μM as large CTCs (Fig. 3, white arrows). We also observed 3D CTC tumorspheres to expand as an endomembrane partitioning-like system (Supplementary Fig. 1) in which the endomembrane furrow separates the daughter and mother cell during cell-division events^18^.

### Biomarker profiling of CTC subsets

**Figure 4:**
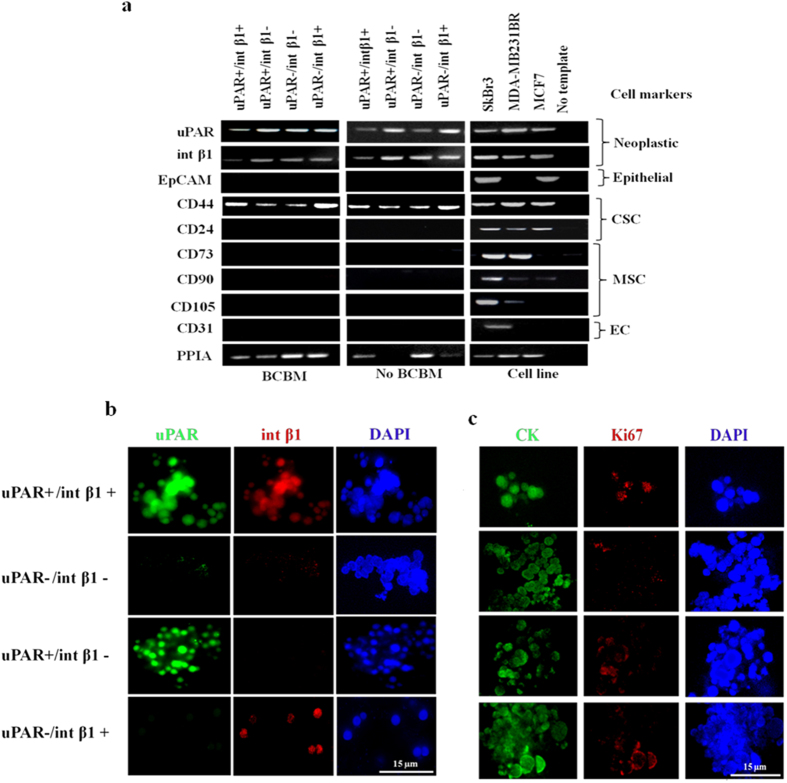
Biomarker profiling of uPAR/int β1 in 3D CTC tumorspheres. (**a**) EpCAM-negative/CD45^−^/CD44^+^/CD24^−^ and uPAR/int β1 CTC subsets were cultured as 3D CTC tumorspheres. mRNAs were amplified by REPLI-g WTA single-cell kit (Qiagen) followed by RT-PCR analyses. Polypropyl isomerase (PPIA) was used as internal loading control. MCF7, MDA-MB-231Br and SKBr3 cell lines were used as additional positive/negative controls. CSC, Cancer Stem-Cell; MSC, Mesenchymal Stem-Cell; EC, Endothelial Circulating Cell; BCBM, Breast Cancer Brain Metastasis. All other data are representation of at least triplicate independent experiments. Full-size gel images are incorporated in Supplementary figure 3; **(b,c)** Immunofluorescence staining was done for combinatorial expression of **(b)** uPAR and int β1 **(c)** pan-cytokeratin and Ki67 markers. Deconvulated cell imaging and projection were done by DeltaVision Deconvolution Microscope (GE Healthcare Life Sciences, Inc.), and analyzed by SoftWoRx software version 6.1.3 (GE Healthcare Life Sciences, Inc.) at 100X. Scale bars, 15 μM. Brightness and contrast of images were adjusted for publication purposes. Representative images are shown.

To validate the specific expression of cell-surface markers used for CTC enrichment, we performed RT-PCR analyses. We amplified mRNAs from 3D CTC tumorspheres obtained from breast cancer patients with or without BCBM, and analyzed them by RT-PCR to assess expression levels of neoplastic (uPAR/int β1), tumor epithelial (EpCAM), circulating endothelial (CD31), mesenchymal stem cell (CD73, CD90 and CD103) and breast cancer stem cell (CD44^+^/CD24^−^) markers. We detected the presence of neoplastic and breast cancer stem cell markers coupled with negativity for EpCAM (Fig. 4a). Next, to confirm that isolated CTCs subsets did not represent non-CTC populations, we evaluated specific transcript levels for the expression of mesenchymal stem cells (CD73, CD90 and CD105) and circulating endothelial (CD31) markers (Fig. 4a). These markers were not expressed in *in vitro* 3D CTC tumorspheres (Fig. 4a). The absence of circulating mesenchymal and endothelial markers suggests that these putative 3D CTCs tumorspheres had a non-hematopoietic origin and that they did not derive from non-CTC populations. Moreover, we assessed *in vitro* 3D CTC tumorspheres to retain original gene expression patterns irrespective of the initial selection under long-term *in vitro* culture conditions. Further, we assessed protein expression of CTC subsets uPAR and int β1 markers by immunofluorescence on *in vitro* 3D CTC tumorspheres. We found that these CTC subsets possessed a characteristic combinatorial expression pattern on their cell-surface (Fig. 4b). Lastly, we verified the neoplastic origin and proliferating abilities of 3D CTC tumorspheres by evaluating the pan-cytokeratin and Ki67 expression and confirmed their detection in uPAR/int β1 3D CTC tumorspheres (Fig. 4c).

### CTC single-cell genotyping

**Figure 5:**
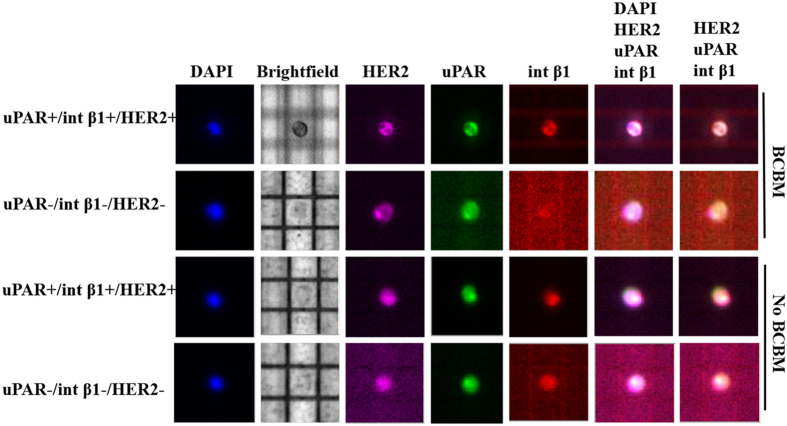
Single-cell DEPArray^™^ isolation of uPAR/int β1 CTC subsets from breast cancer patients. Multiparametric flow cytometry (Six fluorescence channels, ARIA IID system, BD Biosciences^™^) was applied to select EpCAM-negative/CD45^−^/CD44^+^/CD24^−^ CTC followed by DEPArray single-cell isolation to select a combinatorial expression of uPAR (FITC), int β1 (ApC) and human epidermal growth factor receptor-2 (HER-2) (PE). DEPArray^™^ (Silicon Biosystems, Inc.) analyses were subsequently performed by Cell Browser^TM^ software. Representative single CTCs captured and isolated by DEPArray^™^ are shown. DAPI (ThermoFisher Scientific; cat # D1306) = nuclear staining blue. BF = Brightfield.

To dissect the heterogeneity of CTC subsets at a single-cell level, we captured cells positive or negative for uPAR, int β1 and HER2 expression markers using the dielectrophoretic array platform DEPArray^™^ (Silicon Biosystems, Inc.), following a pre-enrichment step of CD45^−^/EpCAM-negative/CD44^+^/CD24^−^ CTCs derived from BCBM and no BCBM patients (Fig. 5). Of note, DEPArray^™^ technology enables the isolation of viable CTCs for interrogation of CTCs on a cell-per-cell basis, the smallest functional unit of cancer^19^. CTC subsets were sorted per DEPArray^™^ specifications (all-or-none threshold for CTC marker expression) employing uPAR, int β1 and HER2 selection. Next, the genomic content of DEPArray™-sorted CTCs containing combinatorial expression of these markers (uPAR^+/−^/int β1^+/−^ and HER2^+/−^) was assessed at single-cell level. Single CTCs were amplified employing the *Ampli1*^™^ WGA method (Silicon Biosystems, Inc.) and mutation analyses of >200 hallmark cancer genes were carried out by applying the MassARRAY^™^ detection system (Sequenom, Inc.) on DEPArray™-sorted single CTCs (n = 7). We were able to detect the presence of HSP90AB1 C2139T, PRKCB G785T, AURKC C154G and JAK2 A2049CT cosmic mutations in BCBM-derived CTCs at the single-cell level, while PRKCB G785T missense mutations were found in CTCs irrespective of expression markers considered and BCBM status (Supplementary table S2).

### Characterization of CTC subsets revealed distinct *in vitro* biological patterns

**Figure 6:**
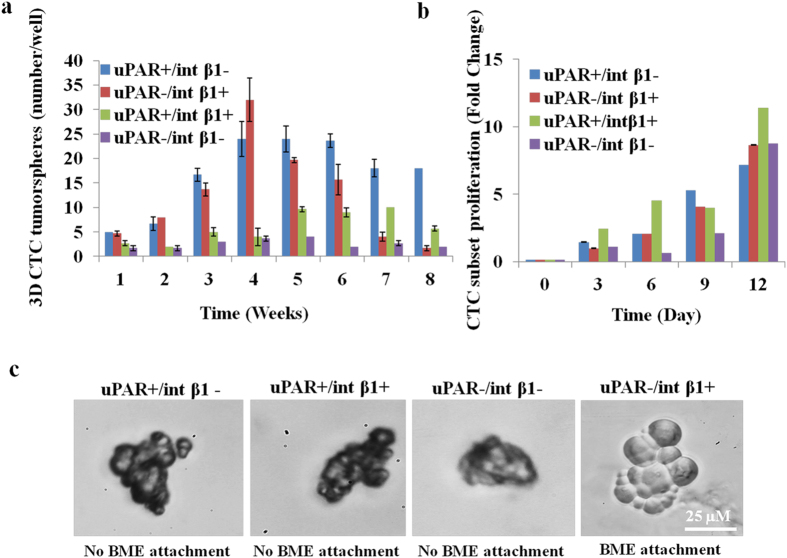
*In vitro* characterization of 3D CTC tumorspheres. **(a)** Generation of CTC tumorspheres over time in culture. Tumorsphere assays were performed in FACS sorted (CD45^−^/CD44^+^/CD24^−^/EpCAM-negative/uPAR^+/−^/int β1^+/−^) *in vitro* 3D CTC subsets derived from no BCBM patient. Trypsinized 10-15 3D CTC tumorspheres were cultured in 96-well plate coated with 1% soft agar and quantified at successive weeks under phase contrast microscopy (Zeiss, Inc.); **(b)** CTCs cell proliferation assays (WST-1, Roche Life Sciences, Inc.) over time in culture were performed in FACS-sorted *in vitro* 3D CTC subsets containing uPAR/int β1 combinatorial expression. Trypsinized 10-15 3D CTC tumorspheres were cultured in 96-well plate coated with 1% soft agar. Absorbance was measured at 450 nm and 690 nm wavelength at 8 hrs after adding WST-1 reagent at different time points. All data are representative of at least three independent experiments with mean standard deviation (±). Student paired type *2 t*-test was performed and *p*-value* (<0.01) were calculated and found to be significant; **(c)** CTC adhesion assays. Four CTC subsets with combinatorial expression of uPAR and int β1 were aliquoted into 96 well flat-bottom plates coated with Trevigen^®^ PathClear Basement Membrane Extract^®^ (BME) and incubated for 96 hours at 37 °C for adhesion assay.

To interrogate 3D CTC subsets for multiple *in vitro* properties as related to steps of the metastatic cascade, we investigated the spatial-temporal kinetics of *in vitro* 3D CTC tumorspheres formation by performing 3D-tumorsphere assays. We observed that uPAR and int β1 combinatorial expression of four CTC subsets expanded in size and number to cluster and generate 3D CTC tumorspheres. Distinct bell-shaped *in vitro* growth patterns were noticeable up to a 10-week analysis endpoint (Fig. 6a; see also Supplementary Fig. 2). Of note, uPAR^+^/int β1^−^ CTC subsets generated 3D CTC macro-tumorspheres (>5 cells) compared to CTC micro-tumorspheres (<5 cells) of uPAR^+^/int β1^+^, uPAR^+^/int β1^−^ and uPAR^−^/int β1^+^ subsets. Conversely, uPAR^−^/int β1^−^ CTC subsets showed delayed clustering and formation of 3D CTC tumorspheres independent of tumorsphere size.

Second, we assessed the proliferative, adhesive and invasive capacities of patient-derived EpCAM-negative CTC subsets. Cell proliferation assays applying 3D non-adherent cells methodologies to 3D CTC tumorspheres revealed that these subsets possessed differential *in vitro* proliferation abilities that correlated with the combinatorial expression of uPAR and int β1 markers. Further, uPAR^+^/int β1^−^ and uPAR^−^/int β1^+^ CTC tumorspheres showed an additive proliferative capacity between days 9 and 12 (Fig. 6b).

**Figure 7:**
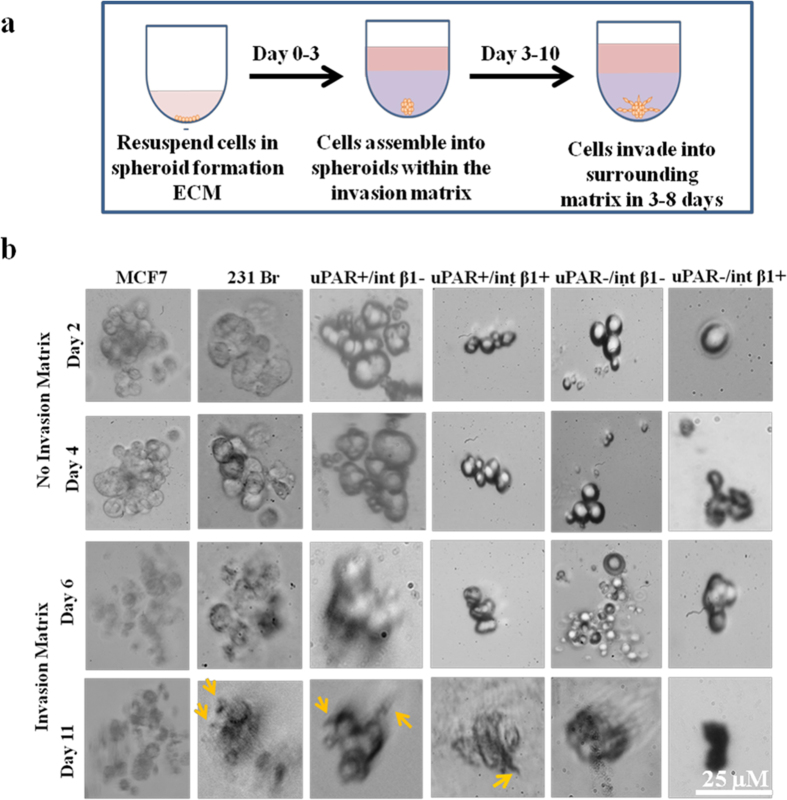
3D invasion assays of *in vitro* 3D CTC tumorspheres. **(a)** Experimental strategy with steps on 3D cell culture 96-well BME cell invasion assays; **(b)** Four CTC tumorspheres with breast cancer no brain metastasis were trypsinized and dissociated as single CTC units or pairlet cells. Control consisted of non-invasive MCF7 and invasive 231Br breast cancer cells. Images were captured at endpoint under 40× magnification using phase contrast microscopy (Zeiss, Inc.). Scale bars, 25 μM. Representative images of three independent experiments are shown.

Third, to evaluate CTC subsets adhesion capabilities, we grew those using Trevigen^®^ basement membrane extract (BME) tumorsphere assays^20^,^21^,^22^. We observed high adhesion of uPAR^−^/int β1^+^ CTC tumorspheres on BME matrix at 48 hours while the other three CTC subsets showed no attachment in adhesion assays even up to 96 hours incubation time (Fig. 6c). Cell migration and invasion are fundamental processes which regulate important cellular events such as angiogenesis, invasion and metastasis of cancer cells. Interestingly, EpCAM-negative CTC subsets aggregated and formed *in vitro* 3D CTC tumorspheres. Accordingly, we determined how CTC tumorspheres generate invadopodia under well‐controlled *in vitro* conditions, capable to become motile and to invade into extracellular matrix (ECM) of the 3D-invasion assay (Fig. 7a). Invadopodia formation by invading CTCs recapitulates the early steps of brain colonization observed *in vivo*^23^. To this end, we assessed Trevigen^®^ 3D tumorsphere invasion assays^20^ on *in vitro* 3D CTC tumorspheres and visualized invadopodia formation. We used non-invasive poorly metastatic MCF7 and highly metastatic 231Br breast cancer cells as negative and positive controls, respectively. We processed invasion matrix to monitor invadopodia formation at day 4. Non-invasive control MCF7 cell-derived spheroids did not form any protrusions whereas invadopodia formation was noted employing invasive 231Br spheroids. Of note, protrusions and tiny ruffle-like invadopodia were observed in uPAR^+^/int β1^−^ and uPAR^+^/int β1^+^ CTC subsets at day 11 (Fig. 7b, yellow arrows). Conversely, no invadopodia formation was observed in uPAR^−^/int β1^−^ and uPAR^−^/int β1^+^ 3D CTC subset spheroids plated on BME invasion matrix per assay specifications^20^. These results demonstrate that the uPAR/int β1 biomarker axis enables invadopodia formation when subjected to the proper tumor microenvironment and factors. They are of relevance because the formation of invadopodia in CTC is required for the *in vivo* extravasation through blood-brain barrier as the early step toward CTC colonization of brain and BCBM development^23^.

Fourth, we confirmed the EpCAM status of *in vitro* 3D CTC tumorspheres by FDA-cleared CellSearch^®^ CTC testing which is however capable to capture only CTCs positive for EpCAM^24^. We spiked ~100 cells of EpCAM-negative *in vitro* 3D CTC tumorsphere cells in blood from normal healthy donors. We were able to capture only 1/100 EpCAM-positive CTCs from CellSearch^®^ analyses (Supplementary table S3). These findings demonstrates that the EpCAM-negative CTC subsets retain their expression under long-term *in vitro* conditions.

### CTC gene expression profiling

**Figure 8:**
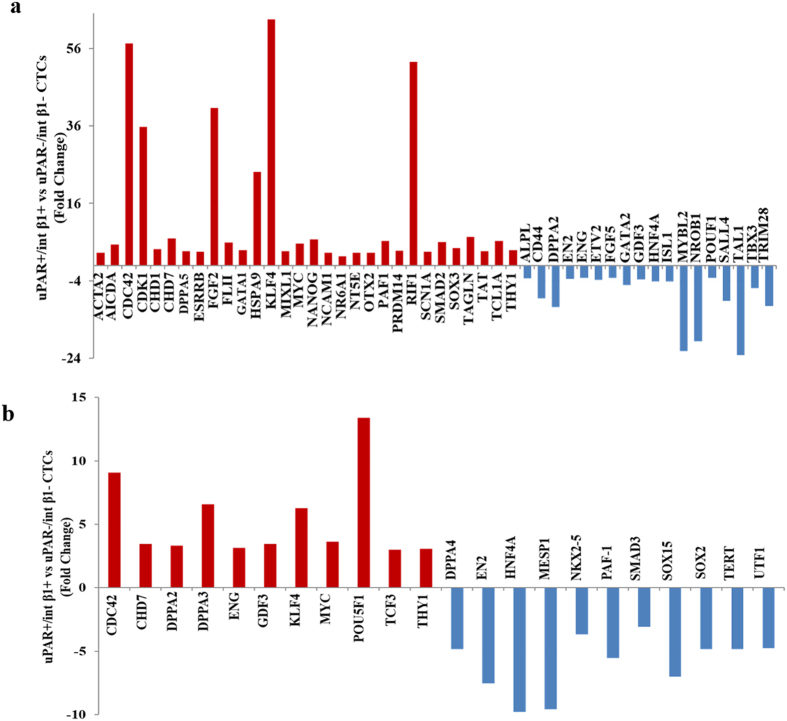
Embryonic stem cell gene expression profiling. PBMCs subpopulation of breast cancer patient with and without brain metastasis were sorted by FACS. uPAR^+/−^ and int β1^+/−^ population were collected respectively (containing EpCAM-negative/CD45^−^/CD44^+^/CD24^−^expression markers). RNA were extracted, amplified and real-time PCR analysis were performed using RT^2^-PCR embryonic stem cell array profiler (Qiagen). The change in mRNA expression (>3 fold) is shown comparing uPAR^+^/int β1^+^ population to uPAR^−^/int β1^−^ population in CTCs isolated from patients clinically diagnosed with BCBM **(a)** or without BCBM **(b).**

CTCs containing stem cell properties undergo embryonic trans-differentiation at distant organs during metastasis. We performed real-time-PCR (RT^2^-PCR) human embryonic stem cell array (Qiagen) profiling to determine the expression of 83 candidate genes in FACS-sorted EpCAM-negative, uPAR^+^/int β1^+^ and uPAR^−^/int β1^−^ stem cell CTC subsets derived from clinically diagnosed breast cancer patient with or without BCBM. Real-time PCR analyses revealed >30 fold increased expression of CDC42, CDK1, FGF2, RIF1, HSPA9 and KLF4 genes between uPAR^+^/int β1^+^ and uPAR^-^/int β1^−^ CTC subsets over the five internal controls of RT^2^ PCR profiler array (Qiagen) and in relation to patient BCBM status (Fig. 8a). Further, CDC42 and POU5F1 gene expression level were relatively higher (>8 fold) when uPAR^+^/int β1^+^ compared with uPAR^−^/int β1^−^ CTC subsets in breast cancer patient without BCBM (Fig. 8b). These findings suggest that uPAR^+^/int β1^+^ CTC subsets possess gene profiles for increased proliferation, DNA damage repair pathway and relate closely to BCBM onset.

## Discussion

CTCs are the “seeds” of uncurable metastasis and can represent a promising and effective alternative to invasive tumor biopsies to detect, monitor and combat solid tumors in patients^3^,^4^,^5^,^6^. However, thus far, only one platform CellSearch^®^ (Janssen Diagnostics, LLC.) has been cleared by the FDA for CTC clinical testing and application. While CellSearch^®^ provided a breakthrough in the CTC field, there are known limitations by this platform since it captures only CTCs positive for the epithelial cell adhesion molecule (EpCAM-positive CTCs)^24^,^25^. Furthermore, CellSearch^®^ involves a fixation step and CTCs captured this way cannot be interrogated further for other downstream application such as RNA-based measurements and culturing CTCs under *in vitro* and *in vivo* conditions. This can be particularly relevant towards discriminating CTC critical for the development of metastasis *vs* ones non metastasis-competent (“irrelevant” CTCs)^4^. These insights have an added impact in breast cancer, a disease known to have high frequency of recurrence following excision of the primary tumor^26^,^27^. We have previously demonstrated that EpCAM-negative CTCs isolated from breast cancer patients were competent for metastasis in xenografts^13^. Further, we have reported identifiers relevant to the breast cancer brain-metastasis-selected CTC profile suggesting their biological and functional relevance in BCBM^13^. Considering the heterogeneity of CTCs, we hypothesized that multiple and contrasting biomarkers are responsible for mechanisms leading to BCBM onset; and additive or alternative to the brain-metastasis selected CTC profile^13^. The purpose of this study was to identify, isolate and characterize CTC subsets with properties related to breast cancer dormancy. We focused on EpCAM-negative CTCs possessing alternative combinations of urokinase plasminogen activator receptor (uPAR) and integrin β1 (int β1), two biomarkers known to be directly implicated in breast cancer dormancy^1^,^2^.

We applied multiparametric flow cytometry and CD45^−^/CD44^+^/CD24^−^ as initial selection markers and specific criteria for EpCAM-positive and EpCAM-negative CTCs: EpCAM-negative PBMCs derived from breast cancer patients sorted through multiparamteric flow cytometry followed by the selection of uPAR/int β1 combinatorial CTC subset expression (Fig. 1). First, gene expression profiling of 83 breast cancer candidates revealed that enriched CTC population disseminate from their primary neoplastic breast tumor and have their unique gene signature (Fig. 2a). Furthermore, the presence of a unique STR DNA fingerprinting of sorted cells revealed their authenticity as putative CTCs which were distinct from human breast cancer cell lines (Fig. 2b). Of note, embryonic stem-cell gene expression profiling revealed the high expression of CDK1, HSPA9, CDC42, FGF2, KLF4 and RIF1 genes in uPAR^+^/int β1^+^ CTC subsets when compared with uPAR^−^/int β1^−^ CTC subsets in BCBM patients (Fig. 8a). FGF2 and KLF4 genes play an important role in blood-brain barrier permeability^28^,^29^, RIF1 is involved in DNA repair pathways^30^ whereas CDK1 and CDC42 are profoundly implicated in mechanisms regulating cell proliferation^31^,^32^. Accordingly, the high expression of above-indicated genes suggests the BCBM competency of uPAR^+^/int β1^+^ CTC subsets additive to the brain metastasis-selected marker profile we have previously discovered^13^.

Second, we were able to grow FACS-sorted CTC populations and to expand them as 3D CTC tumorspheres under *in vitro* conditions (Fig. 3). It was recently reported that CTC clusters derived from primary breast cancer tumor have more metastatic competency compared to single CTCs^33^. Our *in vitro* CTC subsets population expanded as 3D tumorspheres in non-adherent stem-cell conditions; however, they did not fully dissociate when trypsinized suggesting metastatic competency. We also observed cellular protrusions stemming at the periphery of these 3D CTC tumorspheres during *in vitro* expansion (Fig. 3 and Supplementary Fig. 1). Di Vizio *et al.*^34^ found that tumor microvesicles present in the circulation of aggressive form of prostate cancer and their presence in tumor microenvironment may be functionally relevant in potentiating metastasis. Our findings using uPAR/int β1 CTC subsets are consistent with these notions. Thus, elucidating the mechanisms for the generation of tumor-associated vesicles, termed oncosomes, and how they mediate intracellular signaling will be of significance in metastatic breast cancer.

Third, we investigated whether these CTCs subsets retain their initial selective markers uPAR and int β1 under *in vitro* conditions. We observed that the combinatorial expression of uPAR and int β1 remains constant to their selection and were not altered during *in vitro* expansion (Fig. 4a). The lack of mesenchymal (CD90, CD73 and CD105)^35^ and circulating endothelial (CD31)^36^ markers expression in 3D CTC tumorspheres indicate that these putative 3D CTC tumorspheres are non-hematopoietic, tumorigenic, and contain stem-cell properties, eg, presence of the CD44^+^/CD24^−^ axis. Further, positivity of Ki67, cytokeratins (CK) along with EpCAM negativity in 3D CTC tumorspheres (Fig. 4b,c) suggest their hybrid or plastic state required for transition/interchange of mesenchymal to epithelial properties postulated for metastasis to occur^37^.

Disseminated EpCAM-negative CTCs undergo mesenchymal-epithelial transition (MET) at distant organs, invade the tissue and then become localized to generate metastatic tumors. Accordingly, CTC adhesion, proliferation, invasion and tumorsphere formation are of value to characterize CTCs at cellular and molecular levels. The neoplastic markers, uPAR and int β1 interact with each other to drive tumor growth by regulating the cross-talk with the target organs of microenvironments. Interestingly, the ablation of uPAR and int β1 switches the proliferative cell to dormant G_0_-G1 arrest state resulting in tumor suppression *in vivo*^1^,^15^. We observed uPAR^+^/int β1^+^ 3D CTC tumorspheres to be more proliferative compared with CTC populations containing the uPAR^−^/int β1^−^ CTC subsets having this dormancy axis (Fig. 6a,b). Additionally, the presence of invadopodia formation/cell invasiveness in uPAR^+^/int β1^−^ and uPAR^+^/int β1^+^ 3D CTC tumorspheres advocates for their metastatic competency (Fig. 7b). Fourth, uPAR/int β1 CTC subsets underwent expansion in size, volume and number prior to CTC clustering and 3D CTC tumorspheres formation at variable rates *via* an endomembrane partitioning-like system (Supplementary Fig. 1, yellow arrows)^18^. Further experiments with xenografts and live-cell imaging using membrane binding and nuclear dyes will be required to confirm the mechanism of CTC clustering and 3D CTC tumorsphere formation *in vivo*. Regardless, our findings are of significance to clinical dormancy since CTCs shed from the primary tumor exhibit various CTC circulator phenotypes via a mechanism(s) of expansion that are yet unknown. These phenotypes are dependent on the biomarker expression such as presence of uPAR and int β1 axis. Multiple circulator CTC phenotypes must exist; they resist apoptosis, undergo evolution and clonal selection *via* DNA damage and active DNA repair pathways, and avoid arrest and adhesion to target organs with extreme efficiency. Selected CTC clones specific for uPAR/int β1 biomarker axis undergo proliferation and expansion for a long-term niche pool, and CTC clustering for secondary tumorsphere formation. For example, it is known that breast cancer cells grow in a disorganized fashion on reconstituted basement membrane assays by employing int β1 and epidermal growth factor (EGF)-dependent signaling pathways^36^. We observed int β1-dependent adhesion capabilities of dormant tumor populations in uPAR^-^/int β1^+^ 3D CTC tumorspheres when grown on BME matrix (Fig. 6c). This suggests that int β1^+^ “dormant” CTCs might undergo some degree of differentiation but they become non-proliferative in the absence of uPAR expression^1^,^38^.

Lastly, heterogeneous populations of CTCs harbor genetic and epigenetic changes at single-cell level^39^,^40^,^41^,^42^ and exhibit distinct breast cancer phenotypes^43^. We used the DEPArray^™^ platform (Silicon Biosystems, Inc.) to dissect CTCs at single-cell level derived from BCBM *vs* no BCBM followed by MassARRAY^™^ mutation analysis (Sequenom, Inc., Supplementary table S2). We detected common cosmic mutation PRKCB G785T in patient-derived CTCs, regardless of their expression markers (uPAR/int β1/HER2) or brain metastasis clinical status. However, BCBM-derived CTC subsets contained cosmic mutation HSP90AB1 C2139T in uPAR^+^/int β1^+^/HER2^+^ CTC, and AURKC C154G and JAK2 A2049CT mutations in two different CTCs containing uPAR^−^/int β1^–^/HER2^–^ expression. Accordingly, while the variability of genetic mutations at the single-cell CTC level confirmed the high heterogeneity of CTCs, it can provide a better approach to evaluate the biology of CTCs by targeting these mutations and assessing their impact.

In conclusion, the detailed characterization and application of uPAR/int β1 CTC subsets can be useful to decipher cellular and molecular mechanisms of organ-homing CTCs and to better understand breast cancer dormancy *versus* CTCs abilities to adhere, proliferate and invade, which are hallmark properties of tumor progression. This study represents a step forward towards early detection and treatment of breast cancer-associated brain metastasis. The extension of these investigations will be a clinically useful tool in personalized medicine applications for effective drug screening/testing method rather than cellular transplantation.

## Methods

### Patient samples and blood collection

Blood samples were collected from 38 advanced breast cancer patients diagnosed with or without BCBM. This was performed according to a protocol approved by the Institutional Review Board at MD Anderson Cancer Center with patients providing informed consent. Patients were required to have clinical and radiological evidence of progressive breast cancer for their inclusion in this study. Patients underwent systemic therapy as appropriate for their malignancy and irrespective of CTC status. Of the 38 patients with advanced breast cancer (median age of breast cancer patients = 56 years; median number of prior therapies among patients with breast cancer = 5.5), 21 patients were ER/PR positive (55.3%), 10 patients were triple negative (26.3%), and 8 patients were HER2 positive (21.1%). Among the 38 patients with breast cancer, 21 patients (21 of 38 patients, 55.3%) had brain metastasis and 17 patients (17 of 38 patients, 44.7%) did not have brain metastasis (Table 1). Details of each selected patient were provided in the supplementary table S1. Only patients starting a new line of therapy were enrolled in the present study. Patients with concurrent disease(s) were excluded. Peripheral blood (25–45 mls/patient) was obtained at the middle of vein puncture after the first 5 ml of blood was discarded to avoid contamination by normal epithelial cells. All samples (25–45 mls blood) were collected using CellSave^TM^ (Janssen Diagnostics, LLC) or EDTA tubes in sterile conditions according to CTC testing to be performed, and provided immediately to the laboratory for CTC analysis.

### Peripheral blood mononuclear cells isolation

PBMCs were isolated as described elsewhere^44^. Briefly, PBMCs from whole blood were isolated by using red blood cell lysis buffer (154 mM NH4Cl, 10 mM KHCO_3_, 0.1 mM EDTA) at a ratio of 1:25, followed by incubation at room temperature (25 °C) for 5 min, then pelleting remaining blood cells at 300 g for 10 min. Cell pellets, consisting mostly of mononucleated cells, was washed with 20 ml 1X PBS and centrifuged at 300 g for 5 mins. PMBCs were then counted by hemocytometer used for fluorescent labeling and capturing CTC using multi-parametric FACS or other platforms (e.g., CellSearch^®^, DEPArray™, or others).

### CTC selection by FACS

Isolated patient PBMCs were analyzed and sorted by multiparametric flow cytometry (FACS Aria^™^ II lased high-speed flow cytometer, BD Biosciences™) by using DAPI^−^/CD45^−^/EpCAM-negative/CD24^−^/CD44^+^/uPAR^+/−^/int β1^+/−^ selection markers. Between 5.0 × 10^5^ and 2.0 × 10^6^ events were collected per list mode data file and analyzed by DIVA acquisition software version 8 (multiparametric flow cytometry). Antibodies and reagents used were indicated in figure legend (See figure 1).

### CTC subsets culture and growth conditions

FACS-selected CTC populations were grown as tumorsphere using Mammocult^™^ media (StemCell Technologies, Inc.). Enriched CTCs were seeded on 1% agarose in 6-well tissue culture plate. Mammocult^™^ media was then applied and used to grow 3D CTC tumorspheres by incubating cells at 37 °C and 5% CO_2_. 3D CTC tumorspheres were passaged with 0.25% trypsin-EDTA (Gibco Life Technologies, Inc.). CTC subsets were STR DNA fingerprinted (Fig. 2a). They were genetically analyzed by the MassARRAY^™^ detection system (Sequenom, Inc.) to ensure tumor cell fidelity, and periodically assessed for pathogen-free *Mycoplasma* testing. They were used for experimental work only within the first 30–40 days of culture.

### CellSearch^®^ CTC analyses

CellSearch^®^ CTC procedures were applied for 3D CTC tumorsphere analyses. Briefly, approximately 100 cultured cells from each FACS-selected group (uPAR^+^/β1 int^+^, uPAR^+^/int β1^−^, uPAR^−^/int β1^+^ and uPAR^−^/int β1^−^) were spiked into 7.5 ml of peripheral blood from normal donors collected in CellSave^TM^ tubes (Janssen Diagnostics, LLC.) tubes. Samples were loaded onto the CellTracks^®^ AutoPrep. The system added anti-epithelial cell adhesion molecule (EpCAM) ferrofluid to cells. Cells were automatically stained with anti-CK-PE to identify intracellular cytokeratins 8, 18 and 19 with anti-CD45/APC to identify leukocytes and with DAPI to identify cell nuclei^23^,^24^. Finally, samples were loaded onto CellTracks^®^ cartridges for analysis by the CellTracks^®^ Analyzer II. A CTC is defined by CellSearch^®^ as an intact, morphologically round cell with a defined ratio cytoplasm/nuclei that stains positive for CK-PE and DAPI but negative for CD45/APC. CTC enumeration was then determined by one of the authors (W.Y.) who was blinded to all patient data.

### STR DNA fingerprinting

STR DNA fingerprinting was performed in FACS-enriched REPLI-g WGA amplified CTC subsets using the Promega 16 High Sensitivity STR Kit (Cat # DC2100). The STR profiles were compared to online search databases (DSMZ/ATCC/JCRB/RIKEN) of 2455 known profiles; along with 2556 know profiles. The samples were analyzed at Characterized Cell Line Core (CCLC) facility at MD Anderson Cancer Center, Houston, TX.

### Reverse-Transcriptase PCR (RT-PCR)

cDNA was isolated from *in vitro* 3D CTC tumorspheres and amplified by using REPLI-g WTA Single Cell Kit (Qiagen) according to manufacturer instructions protocol. Briefly, cells were lysed followed by gDNA removal. The subsequent reverse transcription reaction was performed by using oligo dT primer to amplify polyA^+^ mRNA enrichment transcripts. The synthesized cDNA was ligated using a high-efficiency ligation mix followed by whole transcriptome amplification of cDNA with the REPLI-g SensiPhi DNA polymerase enzyme. RT-PCR were then performed by using gene specific primers (Supplementary table S4).

### Real-time PCR profiling

Amplified cDNA were purified by ExoSAP-IT (Affymetrix, Inc.) and were subjected to real-time PCR amplification using SYBR green method (Applied Biosystems, Inc.). The relative quantities were measured by five internal controls present in array and were analyzed by RT^2^-PCR profiler array (Qiagen) data analysis software version 3.5.

### DEPArray^™^ CTC analysis

DEPArray^™^ (Silicon Biosystems, Inc.) is a semi-automated technology for detection and isolation of enriched CTCs at single-cell level by dielectrophoresis and CTC visualization at the single-cell level by immunofluroscence staining. CD45^−^/ CD44^+^/CD24^−^/EpCAM-negative FACS-sorted CTC subsets were stained with mouse anti-human uPAR (CD87)-FITC (AbD Serotec, cat # MCA2516488, 1:50 dilution) anti-human int β1 (CD29)-ApC TS2/16 (Biolegend, cat # 3030008, 1:20 dilution) and anti-human HER2–PE (Biolegend, cat # 324405, 1:20 dilution). Subsequently, 14 μl cells were loaded in pre-washed with 325 μl of SB115 buffer (Silicon Biosystems, Inc.) DEPArray^™^ chip (Silicon Biosystems, Inc.) and scanned for detailed characterization of CTCs according to manufacturer’s protocol. The characterized CTCs were collected in a 0.2 ml PCR tube and used for *Ampli1*^™^ WGA amplification.

### *Ampli1* ^™^ WGA amplification

*Ampli*1^™^ WGA procedure (Silicon Biosystem, Inc.) were performed in a single tube according to manufacturer’s protocol. This whole genome amplification method is based on adaptor-ligation-mediated amplification^45^,^46^. Briefly, genomic DNA was digested with MseI restriction enzyme to generate sticky ends fragments followed by ligation of a single adaptor and fill-in reaction. The resultant WGA PCR product (50 μl) was produced by amplification of the entire genome library with one single high specific PCR primer corresponding to the adaptor. The successful amplification of WGA products were analyzed by *Ampli1*^™^ QC kit (Silicon Biosystem, Inc.) according to instructions from the manufacturer.

### DNA mutation analyses

DEPArray^™^- sorted CTCs *Ampli1*^™^ WGA products were purified by DNA mini kit (Qiagen) to analyze >200 mutation of hallmark cancer genes through MassARRAY^™^ detection system (Sequenome, Inc.)^47^. This was performed at Characterized Cell Line Core (CCLC) facility at MD Anderson Cancer Center, Houston, TX.

### Immunofluorescence

FACS-enriched and cultured 3D CTC tumorspheres were fixed with 4% paraformaldehyde and air-dried. Cells were incubated with primary conjugated antibody (1:10 dilution in 5% BSA, 0.5% Tween-20 in 1 X PBS) for 1 hour at room temperature (25 °C). Cells were then washed at least 3-4 times with Cell Staining Buffer (BioLegend^®^, cat # 420201) after each subsequent step. Slides were mounted with DAPI containing mounting media (Vectashield, Vector laboratories Ltd.) and carefully sealed. Fluorescent images were taken by the DeltaVision Deconvolution Microscope (GE Healthcare Life Sciences, Inc.) and analyzed by SoftWoRx software version 6.1.3 (GE Healthcare Life Sciences, Inc.).

### CTC proliferation assays

3D CTC tumorspheres containing about 500 cells were grown in 96-well at different time points. Cell proliferation assays were performed by incubating cells with tetrazolium salt WST1 (Roche Life Technologies, Inc.) for 8 hrs and OD were measured at absorbance 450 nm and 600 nm. Student paired, type 2 *t*-test was applied to calculate *p*-value for statistical significance in between CTC subsets containing combinatorial expression of uPAR and int β1 at different time points.

### 3D CTC tumorsphere growth assays

3D CTC tumorspheres were dissociated as single CTC units or pairlets and then scored using hemocytometer and confirmed for cell viability using 1:1 Trypan Blue (Gibco Life Technologies, Inc.). Twenty-four well flat-bottom plates were coated with 1% soft agar and approximately 10-35 trypsinized CTC units/subset were suspended in 100 μl of Mammocult^™^ (StemCell Technologies, Inc.) media were added in each well in multiples. The tissue culture plate was then incubated at 37 °C to analyze the spatial-temporal kinetics of 3D CTC tumorsphere formation with a 10 week period. CTC growth rate was observed under 10X magnification and images were captured and analyzed every week under 40X magnification using phase-contrast microscopy (Zeiss, Inc.).

### BME adhesion assays

CTC subsets were aliquoted into 96 well flat-bottom tissue culture plates coated with Cultrex^®^ Basement Membrane Extract, PathClear^®^ (BME) (Trevigen^®^, Inc.) and incubated for 96 hours at 37 °C. Plates were then analyzed every 24 hrs for *in vitro* 3D CTC tumorsphere BME adhesion using 10X magnification. Images were captured at 96 hrs endpoint under 40X magnification using phase-contrast microscopy (Zeiss, Inc.).

### 3D spheroid BME cancer cell invasion assays

CTC subsets were dissociated using 0.25% trypsin as single CTC units or pairlets. CTCs were scored using hemocytometer and confirmed for cell viability using 1:1 Trypan Blue (Gibco Life Technologies, Inc.). Dissociated CTCs were detected as per the protocol provided by the Trevigen^®^ assay kit (Trevigen^®^, Inc.)^20^. Approximately 15–25 trypsinized CTC units/subset were suspended in 40 μl of Mammocult^™^ media and 10 μl of 1 × 3D spheroid ECM was mixed well and a total volume of 50 μl added to each well in triplicates in 96 well round-bottom plates provided by the kit. Non-invasive MCF7 and highly invasive 231Br breast cancer cells were used, and cell viability was confirmed after trypsinization. Approximately 10^3^ cells in 40 μl of growth media and 10 μl of 1 × 3D spheroid ECM were mixed well and a total volume of 50 μl was added to each well. The tissue culture plate was incubated at 37 °C for monitoring the 3D CTC tumorspheres formation under microscope with an endpoint of day 4. The images were captured and analyzed every two days under 40× magnification using phase contrast microscopy (Zeiss, Inc.). The invasion matrix was added into each well at day 4 and incubated for 1 hr to gel as per assay protocol. 100 μl of growth media was added to each well and the plate was put in 37 °C incubator for regular monitoring of invadopodia formation from until day 11. Images were captured and analyzed every two days under 40X magnification using phase-contrast microscopy (Zeiss, Inc.).

## Additional Information

**How to cite this article**: Vishnoi, M. *et al.* The isolation and characterization of CTC subsets related to breast cancer dormancy. *Sci. Rep.* **5**, 17533; doi: 10.1038/srep17533 (2015).

## Supplementary Material

Supplementary Information

## Figures and Tables

**Table 1:** Demographic and clinical characteristics of patients with advanced breast cancer.

Clinical Characteristics	Patients with Advanced Breast Cancer median or n (%)
Patients with brain metastasis	21 (55.3%)
Age	56 years
Number of prior therapies	5.5
**Mutations, n (%)**	
ER/PR positive	21 (55.3%)
HER2 positive	8 (21.1%)
Triple negative	10 (26.3%)
