# Supplementary material for: The isolation and characterization of CTC subsets related to breast cancer dormancy
Source: Sci Rep. 2015 Dec 3;5:17533. doi: 10.1038/srep17533 (PMC4668355; doi:10.1038/srep17533)
Supplement: Supplementary Information [file srep17533-s1.doc]

**The isolation and characterization of CTC subsets related to breast cancer dormancy**

Monika Vishnoi^1^, Sirisha Peddibhotla^2^, Wei Yin^1^, Antonio Scamardo^3^, Goldy C. George^3^, David S. Hong^3^, Dario Marchetti^1,4^

^1^ Biomarker Research Center, Houston Methodist Research Institute, Houston, TX

^2^ Pathology & Immunology, Baylor College of Medicine, Houston, TX

^3^ Department of Investigational Cancer Therapeutics, The University of Texas MD Anderson Cancer Center, Houston, TX

^4^ Department of Molecular & Cellular Biology and The Dan L. Duncan Cancer Center, Baylor College of Medicine, Houston, TX

Correspondence and requests for materials should be addressed to Dario Marchetti (e-mail: dmarchetti@houstonmethodist.org).

**Supplementary Information**

**
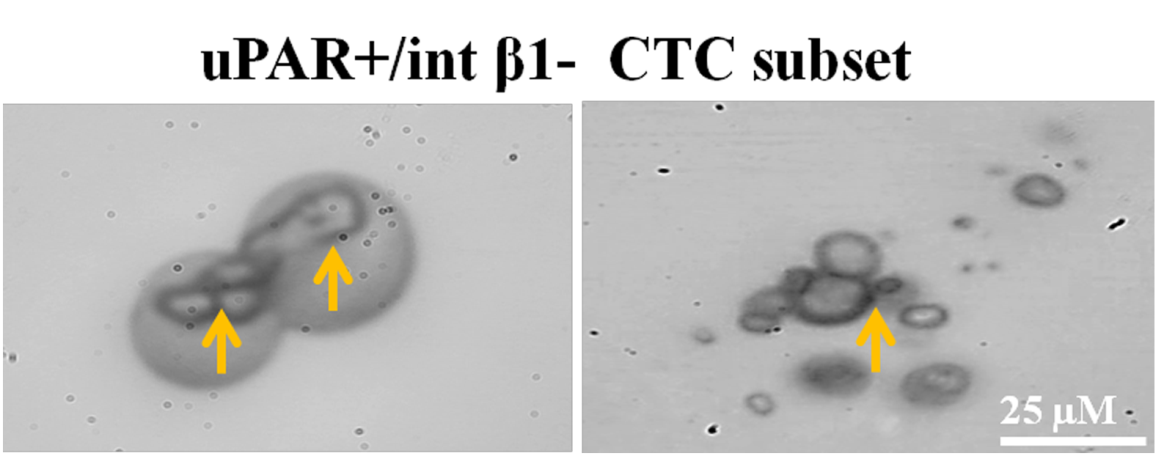
**

**Supplementary Figure 1 | Growth and expansion of uPAR^+^/int β1^-^ subset on 1% soft agar *in vitro* 3D CTC tumorspheres formation assay.** Trypsinized uPAR^+^/int β1^-^ 3D CTC tumorspheres derived from a patient diagnosed with no BCBM undergo growth in size, expand in number *via* an endomembrane partitioning-like system (yellow arrows), and cluster on 1% soft agar to generate *in vitro* 3D CTC tumorspheres in 8 weeks.


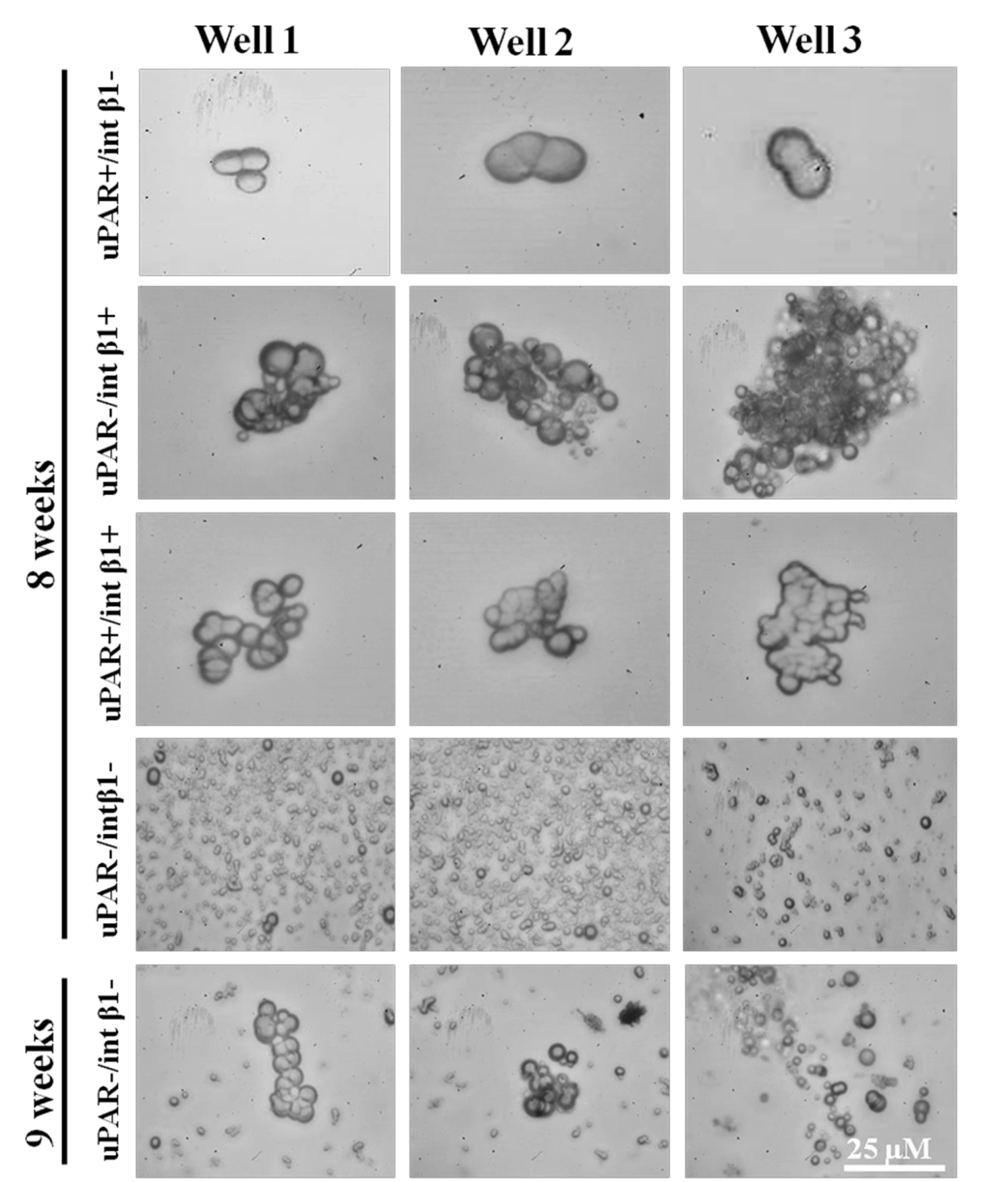


**Supplementary Figure 2 | Spatial and temporal kinetics of *in vitro* 3D CTC tumorspheres**. FACS-sorted EpCAM-negative/CD45^-^/CD44^+^/CD24^-^/uPAR^+/-^/int β1^+/-^ and *in vitro* 3D CTC tumorspheres derived from breast cancer patients were trypsinized according to procedures described in “Methods”. 3D CTC tumorspheres (~10-15 clusters) were cultured on 1% soft agar coated 96-well plate. Images shown were taken at successive weeks using phase contrast microscopy (Zeiss, Inc.).


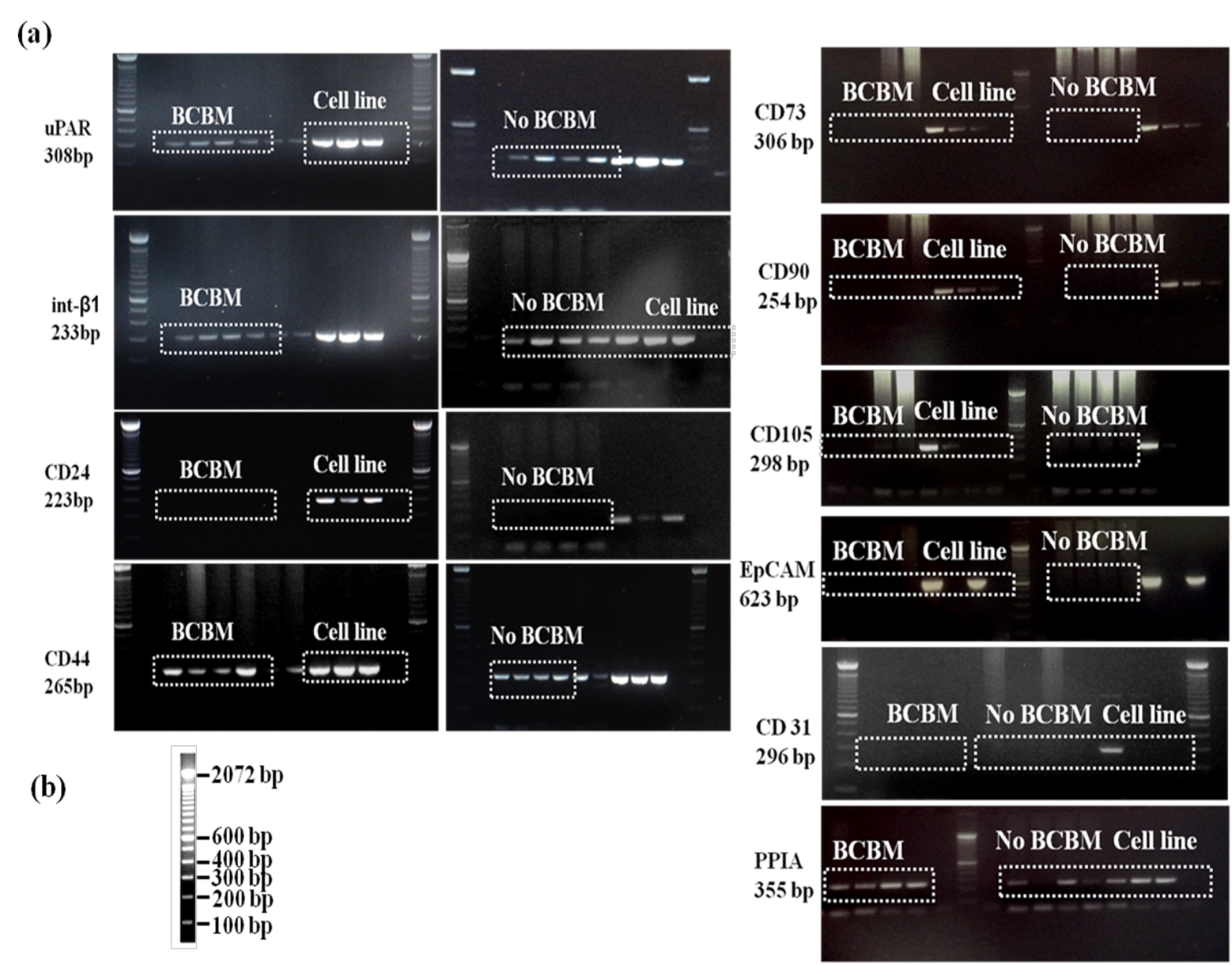


**Supplementary Figure 3 | Figure related to 4a. (a)** Full size agarose gel images showing biomarker profiling of 3D CTC tumorspheres by RT-PCR. White boxes show gel bands of respective genes per panel; **(b)** The 100bp DNA ladder (Life Technologies, cat # 15628-019) which was used for this study.

| **Supplementary Table S1 | Clinical parameters of breast cancer patients analyzed in this study** | | | | | | | | |
| --- | --- | --- | --- | --- | --- | --- | --- | --- |
| **Patient ID** | **Clinical Status** | **Primary cancer** | **HER-2** | **ER** | **PR** | **Diagnostic Stage** | **Age** | **Number of prior therapies** |
| 2 | BCBM | Breast | Positive | Positive | Positive | 4 | 35 | 7 |
| 6 | BCBM | Breast | Negative | Positive | Positive | 4 | 62 | 8 |
| 7 | BCBM | Breast | Negative | Positive | Positive | 4 | 51 | 8 |
| 8 | BCBM | Breast | Negative | Positive | Positive | 3 | 41 | 9 |
| 1 | BCBM | Breast | Positive | Positive | Positive | 3 | 54 | 5 |
| 9 | No BCBM | Breast | Negative | Negative | Negative | 2 | 50 | 3 |
| 11 | BCBM | Breast | Negative | Positive | Positive | 2 | 54 | 10 |
| 12 | BCBM | Breast | Negative | Positive | Positive | 3 | 63 | 9 |
| 13 | No BCBM | Breast | Negative | Negative | Negative | 3 | 66 | 2 |
| 14 | BCBM | Breast | Negative | Positive | Negative | 3 | 37 | 1 |
| 15 | BCBM | Breast | Negative | Negative | Negative | 3 | 46 | 3 |
| 16 | BCBM | Breast | Positive | Positive | Positive | 3 | 45 | 4 |
| 17 | No BCBM | Breast | Negative | Positive | Positive | 4 | 69 | 8 |
| 18 | No BCBM | Breast | Positive | Negative | Negative | 3 | 65 | 2 |
| 19 | BCBM | Breast | Negative | Positive | Negative | 4 | 54 | 4 |
| 20 | No BCBM | Breast | Negative | Negative | Negative | 2 | 68 | 5 |
| 21 | No BCBM | Breast | Negative | Positive | Negative | 3 | 71 | 6 |
| 22 | No BCBM | Breast | Negative | Negative | Negative | 3 | 60 | 4 |
| 23 | No BCBM | Breast | Negative | Positive | Positive | 3 | 50 | 4 |
| 24 | No BCBM | Breast | Negative | Positive | Positive | 2 | 60 | 5 |
| 25 | No BCBM | Breast | Positive | Positive | Positive | 3 | 50 | 6 |
| 26 | BCBM | Breast | Negative | Positive | Negative | 2 | 56 | 6 |
| 27 | No BCBM | Breast | Negative | Negative | Negative | 2 | 65 | 4 |
| 28 | No BCBM | Breast | Negative | Positive | Positive | 2 | 65 | 7 |
| 29 | BCBM | Breast | Positive | Positive | Negative | 4 | 50 | 8 |
| 30 | BCBM | Breast | Negative | Positive | Negative | 2 | 65 | 13 |
| 31 | No BCBM | Breast | Negative | Positive | Positive | 2 | 69 | 6 |
| 32 | BCBM | Breast | Negative | Positive | Positive | 2 | 62 | 6 |
| 33 | No BCBM | Breast | Negative | Negative | Negative | 2 | 27 | 6 |
| 34 | BCBM | Breast | Negative | Negative | Negative | 2 | 68 | 3 |
| 35 | BCBM | Breast | Negative | Positive | Positive | 2 | 43 | 2 |
| 36 | No BCBM | Breast | Negative | Positive | Positive | 2 | 64 | 5 |
| 37 | No BCBM | Breast | Negative | Negative | Negative | 2 | 50 | 4 |
| 38 | BCBM | Breast | Negative | Positive | Positive | 3 | 31 | 4 |
| 39 | BCBM | Breast | Negative | Positive | Positive | 4 | 56 | 9 |
| 40 | BCBM | Breast | Negative | Negative | Negative | 3 | 58 | 8 |
| 41 | BCBM | Breast | Positive | Positive | Positive | 3 | 42 | 8 |
| 42 | No BCBM | Breast | Negative | Positive | Positive | 3 | 64 | 2 |

| **Supplementary Table S2 | Mutation analyses of FACS-enriched, DEPArray™-sorted single CTCs by MassARRAY System™ (Sequenom, Inc.)** | | | | |
| --- | --- | --- | --- | --- |
| **Clinical Status** | **Sample**  **ID** | **CTC**  **genotype** | **Gene Heterozygous Mutation** | **Amino acid Change** |
| BCBM | CTC 1 | uPAR^+^/int β1^+^/Her2^+^ | HSP90AB1 C2139T | G713G |
| BCBM | CTC 2 | uPAR^+^/int β1^+^/Her2^+^ | PRKCB G785T | G262V |
| BCBM | CTC 3 | uPAR^-^/int β1^-^/Her2^-^ | AURKC C154G | H52D |
| BCBM | CTC 4 | uPAR^-^/int β1^-^/Her2^-^ | JAK2 A2049CT | R683S |
| BCBM | CTC 5 | uPAR^-^/int β1-/Her2^-^ | PRKCB G785T | G262V |
| No BCBM | CTC 6 | uPAR^+^/int β1^+^/Her2^+^ | PRKCB G785T | G262V |
| No BCBM | CTC 7 | uPAR^-^/int β1^-^/Her2^-^ | PRKCB G785T | G262V |

**
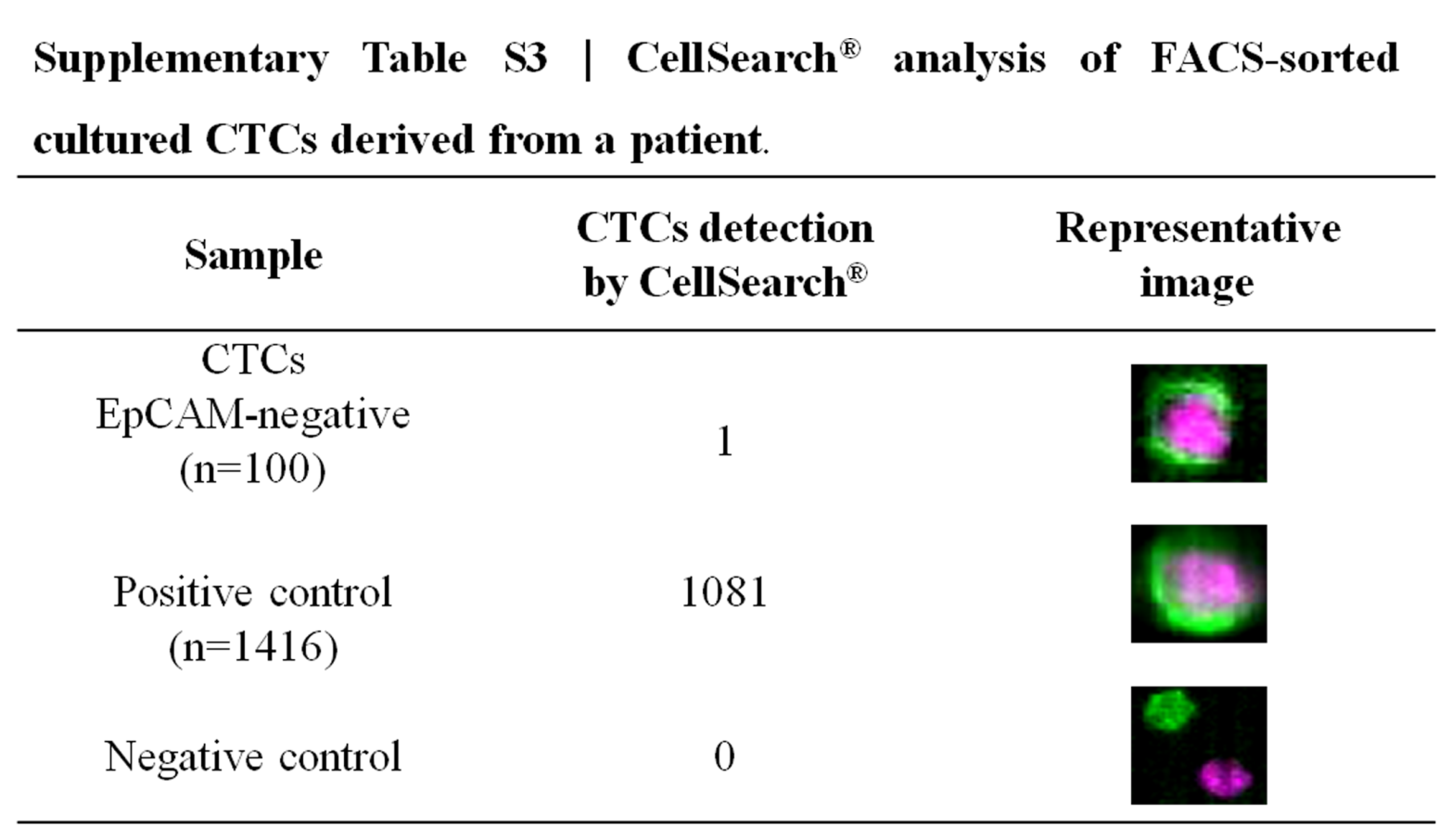
**

| **Supplementary Table S4 | Primer sequences used for gene specific amplification of EpCAM-negative CTC subsets** | | | |
| --- | --- | --- | --- |
| **Gene** | **Primer Sequence**  **(Forward)** | **Primer Sequence**  **(Reverse)** | **Product length (bp)** |
| **EpCAM** | GCTTTATGATCCTGACTGCG | CAGCCTTCTCATACTTTGCC | 623 |
| **CD 44** | TTTGAATATAACCTGCCGCTTTG | GGTGTTGGATGTGAGGATGT | 265 |
| **CD 24** | TCGTGGTCTCACTCTCTCTT | GTTGCCTCTCCTTCATCTTGTA | 292 |
| **uPAR** | GATGCTCCTCTGAAGAGACTTT | CCACAGTCTGGCAGTCATTA | 308 |
| **int β1** | CAAGGTAGAAAGTCGGGACAAA | GCAGTAATGCAAGGCCAATAAG | 233 |
| **CD 31** | CTGAGGGTGAAGGTGATAGC | AGTATTTTGCTTCTGGGGAC | 296 |
| **CD 73** | GCCTGGGAGCTTACGATTT | CAGTCCTTCCACACCATTATCA | 306 |
| **CD 90** | TTCAAATGGAGGGTGTCTCTG | GACCACCGGTTCTTGTAAGT | 254 |
| **CD 105** | AACATGGACAGCCTCTCTTTC | TGGGTATGGGTACTGTGTAGAA | 298 |
| **PPIA** | GAGCACTGGAGAGAAAGGATTT | GGTGATCTTCTTGCTGGTCTT | 355 |
